# Supplementary material for: Environmental enrichment improves social isolation-induced memory impairment: The possible role of ITSN1-Reelin-AMPA receptor signaling pathway
Source: PLoS One. 2024 Jan 19;19(1):e0294354. doi: 10.1371/journal.pone.0294354 (PMC10798460; doi:10.1371/journal.pone.0294354)
Supplement: S1 File — (DOC) [file pone.0294354.s001.doc]

***Supplementary Informations***

**Environmental Enrichment Improves Social Isolation-induced Memory Impairment:  The Possible Role of ITSN1-Reelin-AMPA Receptor Signaling Pathway**

Swamynathan Sowndharya and Koilmani Emmanuvel Rajan*

Behavioural Neuroscience Laboratory

Department of Animal Science,

Bharathidasan University, Tiruchirappalli -620024

India.

*Corresponding Author

Koilmani Emmanuvel Rajan

Behavioural Neuroscience Laboratory

Department of Animal Science,

Bharathidasan University, Tiruchirappalli -620024, India

Email: [emmanuvel1972@yahoo.com](mailto:emmanuvel1972@yahoo.com)

**
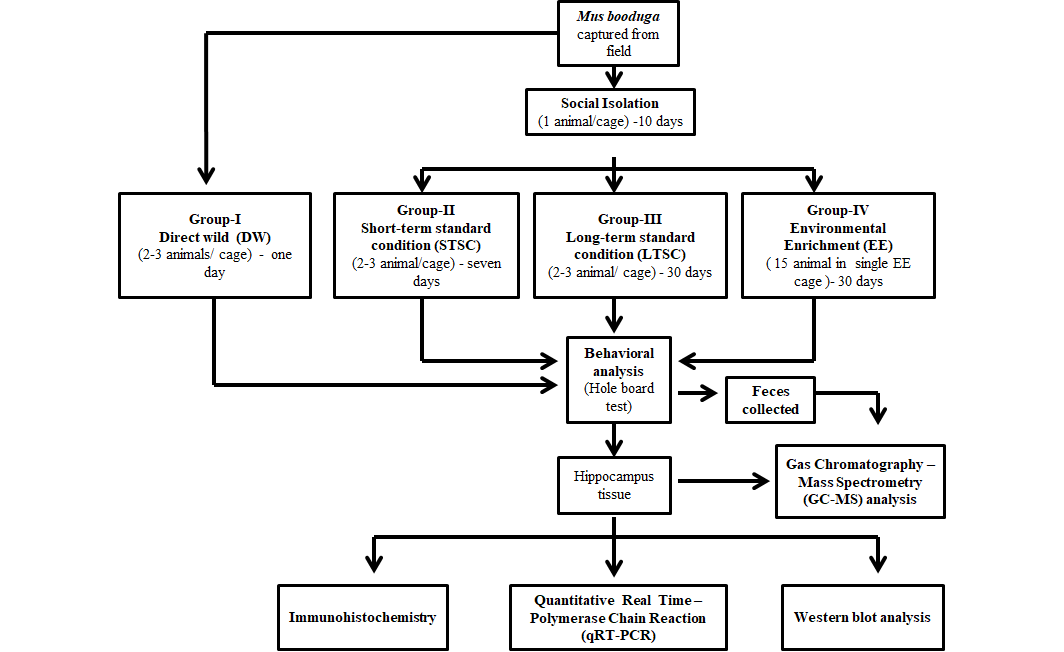
**

**Supplementary figure 1.** Flowchart that showing the complete process of an experimental design. Indian field mice *Mus booduga* were captured with rodent trapper from agricultural field and transferred to Institutional animal maintenance facility. On day 2, (i) Direct Wild (DW) group animals were subjected to hole-board test (HBT). Remaining animals were individually housed for social isolation (SI) in small standard laboratory cage (29.5×22×13cm) for ten days. After the period of SI, animals were randomly divided into three groups : (ii) Short-Term at Standard Condition (STSC), housed at standard laboratory cage for seven days and then subjected to HBT; (iii) Long-Term at Standard Conditions (43×27×15cm), which remained in the laboratory cage for another thirty days, then subjected to HBT. (iv) Environmental Enriched (EE) group [n=15 in EE cage] housed for thirty days and then subjected to HBT. After completing the behavioural test, animals were sacrificed for further analysis.

**
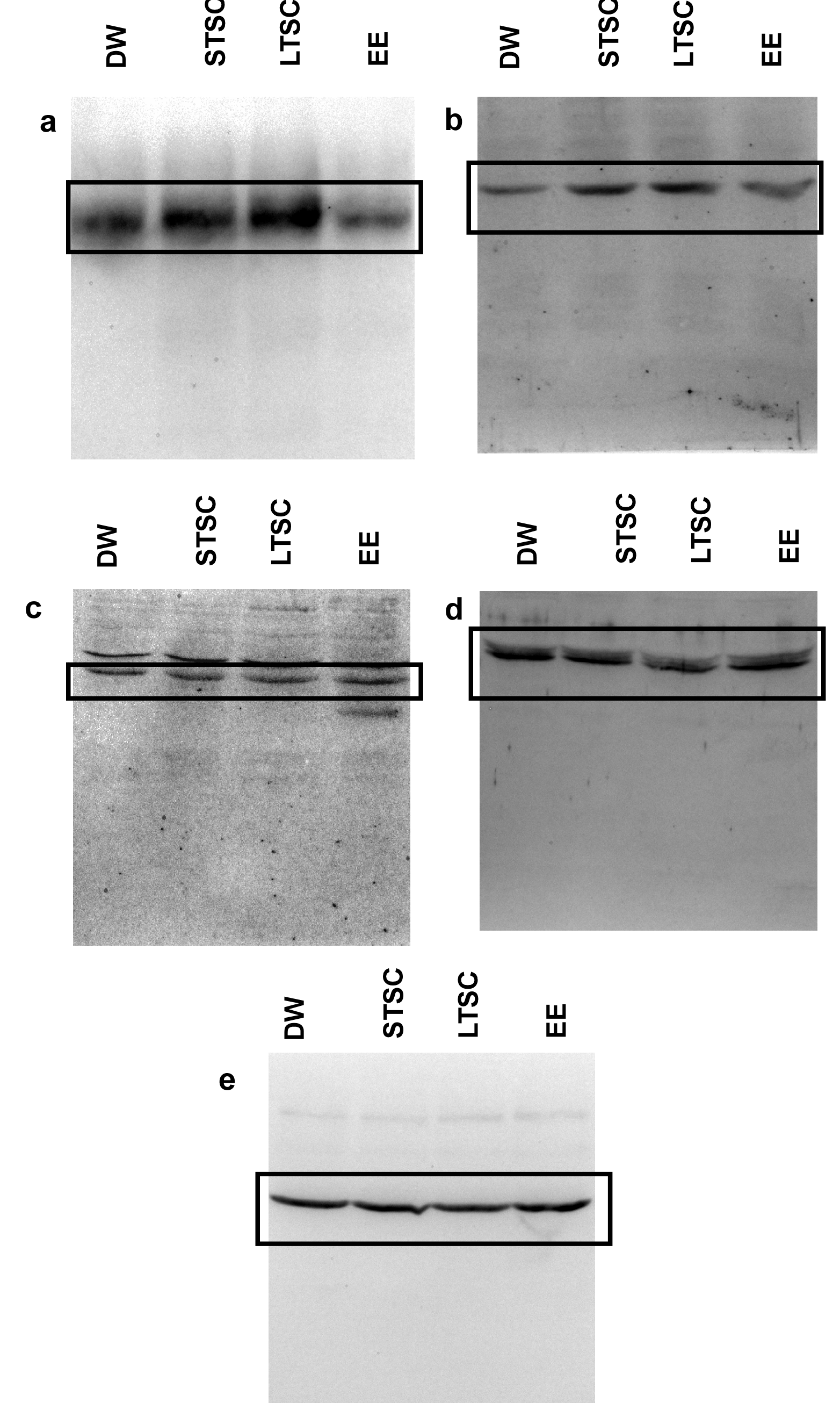
.**

**Supplementary figure 2.** Full immunoblot (uncropped) (a) Intersectin 1 (ITSN1), (b) Huntingtin (Htt), (c) Synaptotagmin 1 (SYT1), (d) Brain-derived neurotrophic factor (BDNF), (e) β-actin used for figure 2 in the manuscript. Gray rectangles are the images cropped from each blot that are shown in the manuscript and each lane representing experimental groups (DW: Direct Wild; STSC: Short-Term at Standard Condition; LTSC: Long-Term at Standard Condition; EE: Environmental Enrichment).

**
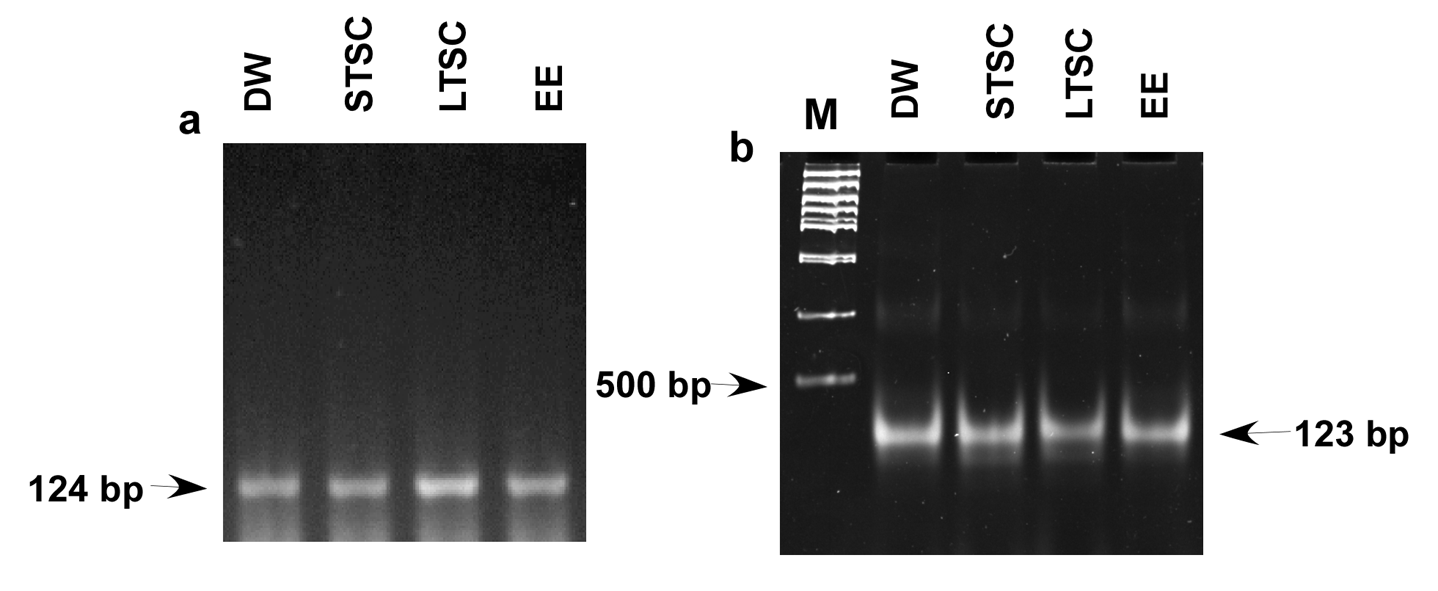
**

**Supplementary figure 3.** Ethidium bromide stained agarose gel showing the mRNA level of (a) *Bdnf* variant III, (b) *Bdnf* variant IV amplified from experimental groups (DW: Direct Wild; STSC: Short-Term at Standard Condition; LTSC: Long-Term at Standard Condition; EE: Environmental Enrichment). Real-time PCR product is supporting for figure 3 in the manuscript.


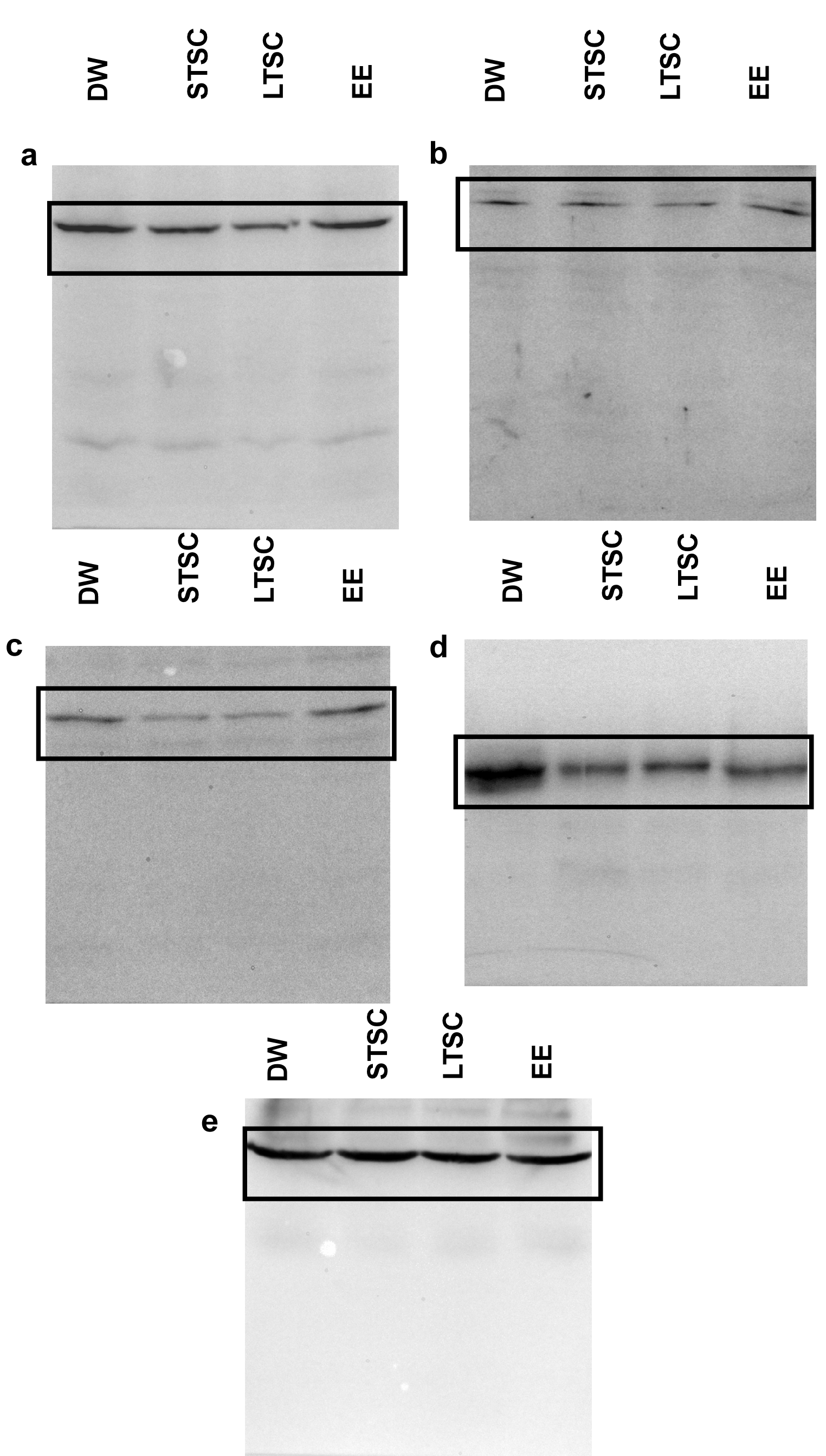


**Supplementary Figure 4.** Full immunoblot (uncropped) (a) Reelin, (b) Apolipoprotein E receptor 2 (ApoER2), (c) Very low-density lipoprotein receptor (VLDLR), (d) Src family tyrosine kinase (SFKs), (e) β-actin used for figure 5 in the manuscript. Gray rectangles are the images cropped from each blot that are shown in the manuscript and each lane representing experimental groups (DW: Direct Wild; STSC: Short-Term at Standard Condition; LTSC: Long-Term at Standard Condition; EE: Environmental Enrichment).

**
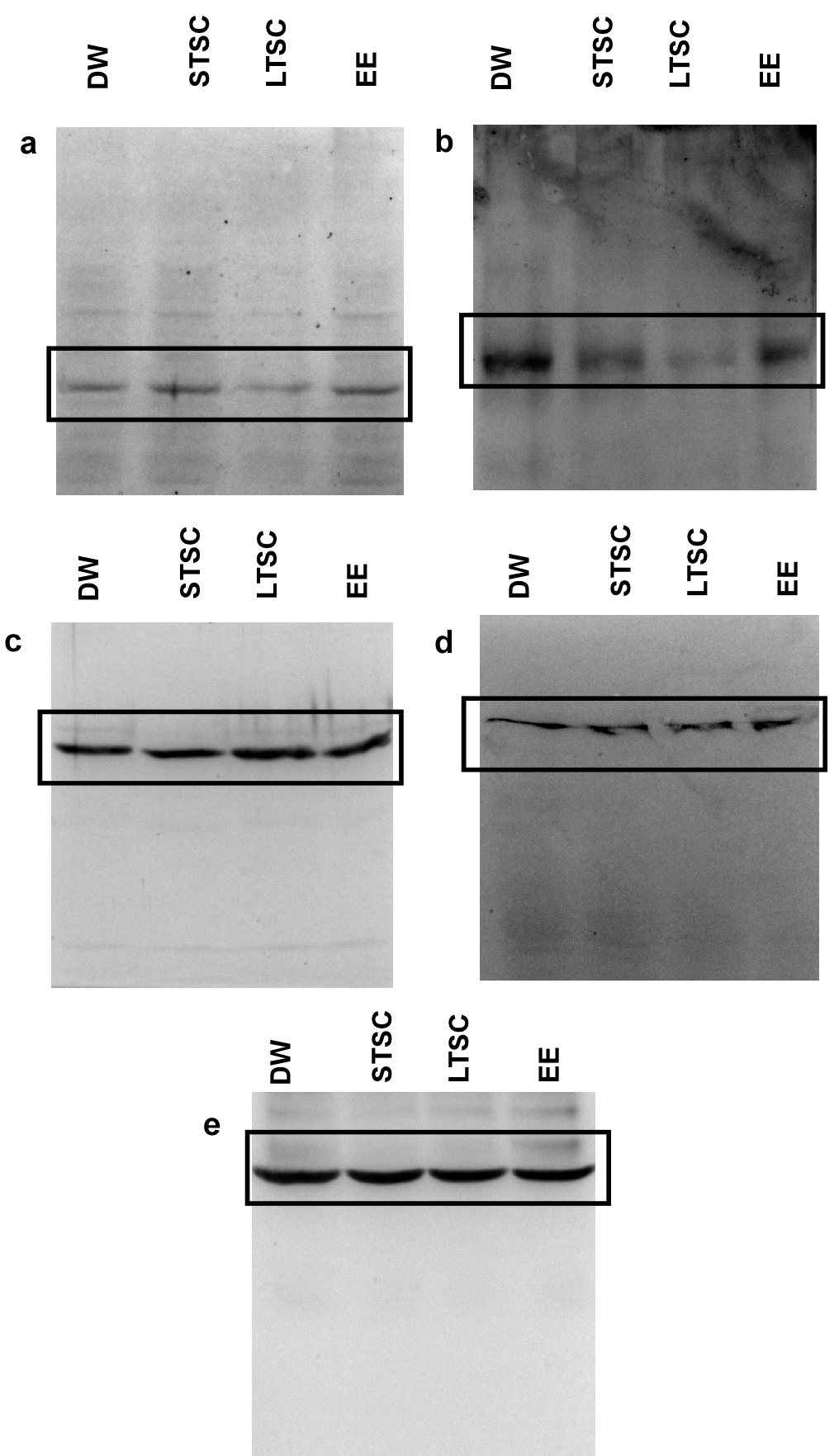
.**

**Supplementary figure 5.** Full immunoblot (uncropped) (a) Disabled protein (Dab)-1, (b) Phosphorylated Dab-1, (c) Protein kinase B (PKB/Akt), (d) Phosphorylated Akt, (e) β-actin used for figure 6 in the manuscript. Gray rectangles are the images cropped from each blot that are shown in the manuscript and each lane representing experimental groups (DW: Direct Wild; STSC: Short-Term at Standard Condition; LTSC: Long-Term at Standard Condition; EE: Environmental Enrichment).


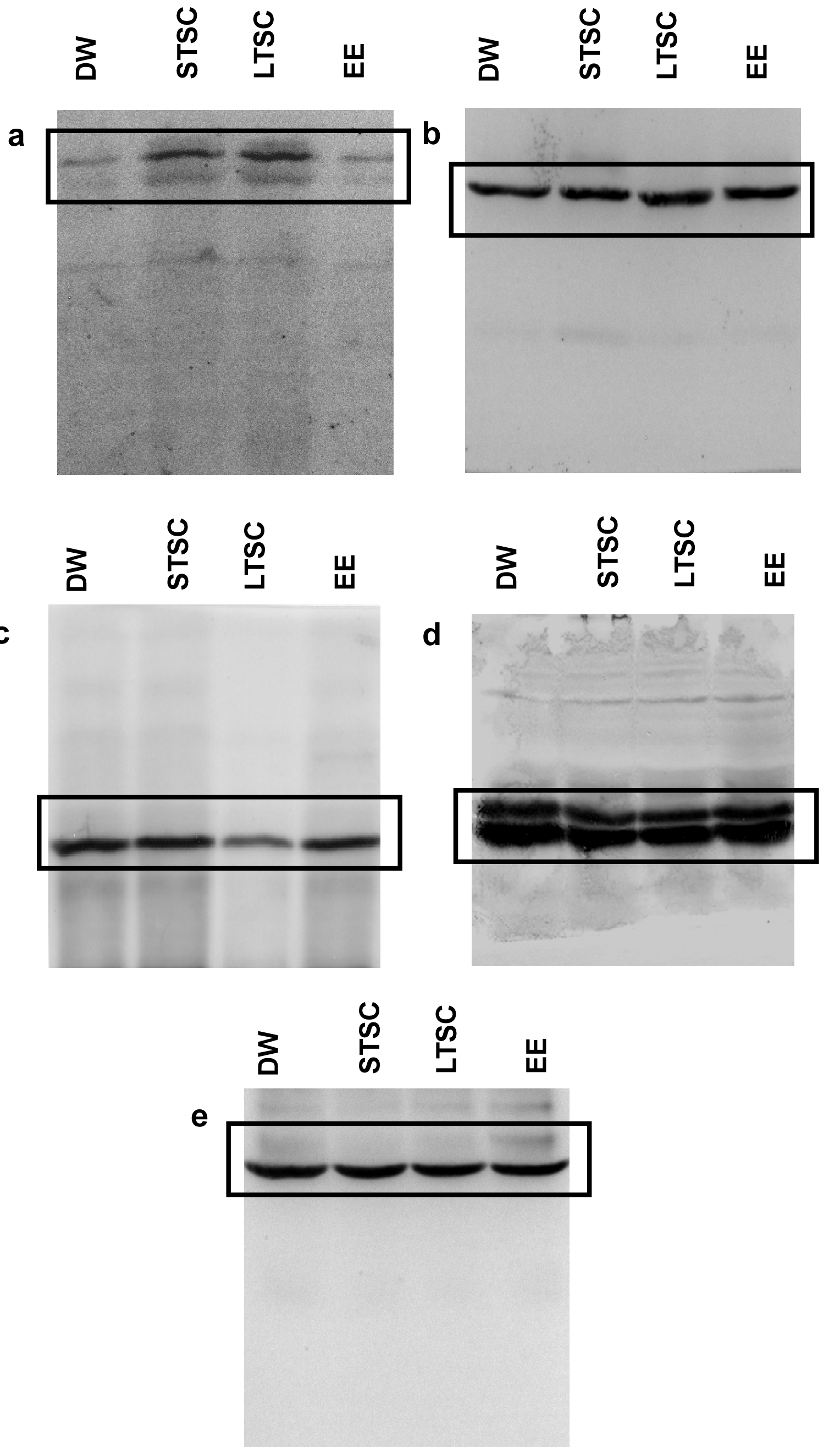


**Supplementary figure 6.** Full immunoblot (uncropped) (a) α-Amino-3-hydroxy-5-methyl-4-isoxazolepropionic acid (AMPA) receptor (GluR1), (b) GluR2, (c) Mitogen-activated protein kinase (MAPK), (d) Extracellular signal-regulated kinase (ERK1/2), (e) β-actin (shown in previous figure) for manuscript figure 7. Gray rectangles are the images cropped from each blot that are shown in the manuscript and each lane representing experimental groups (DW: Direct Wild; STSC: Short-Term at Standard Condition; LTSC: Long-Term at Standard Condition; EE: Environmental Enrichment)

**Minimal Data Set:**

| **Fig. #** | **Mean** | **S.D** | **S.E** | **Statistical method used** | **P value** | **# samples** |
| --- | --- | --- | --- | --- | --- | --- |
| **Fig. 1a** |  |  |  |  |  |  |
| DW – Day 1 | 111 | 41.5612 | 13.14281 | Two Way Analysis of Variance/ Bonferroni t-test | **P˂0.01  ***P˂0.001 | 10 |
| STSC – Day 1 | 144 | 48.72782 | 15.40909 |  |  | 10 |
| LTSC – Day 1 | 186 | 39.96943 | 12.63944 |  |  | 10 |
| EE – Day 1 | 74 | 57.88593 | 14.94608 |  |  | 15 |
| DW – Day 2 | 93 | 56.72703 | 17.93866 |  |  | 10 |
| STSC – Day 2 | 127 | 56.73682 | 17.94176 |  |  | 10 |
| LTSC – Day 2 | 154 | 55.83507 | 17.6566 |  |  | 10 |
| EE – Day 2 | 61 | 32.38048 | 8.360603 |  |  | 15 |
| DW – Day 3 | 74 | 46.12543 | 14.58614 |  |  | 10 |
| STSC – Day 3 | 94 | 33.64537 | 10.6396 |  |  | 10 |
| LTSC – Day 3 | 136 | 48.50659 | 15.33913 |  |  | 10 |
| EE – Day 3 | 44 | 33.2265 | 8.579044 |  |  | 15 |
| DW – Day 4 | 65 | 28.28034 | 8.94303 |  |  | 10 |
| STSC – Day 4 | 71 | 20.27039 | 6.410062 |  |  | 10 |
| LTSC – Day 4 | 118 | 27.46311 | 8.684597 |  |  | 10 |
| EE – Day 4 | 14 | 24.55451 | 6.339946 |  |  | 15 |
| **Fig. 1b** |  |  |  | One way ANOVA/ Bonferroni | *P˂0.05  ***P˂0.001 |  |
| DW | 1 | 0 | 0 |  |  | 10 |
| STSC | 0.755622 | 0.106429 | 0.033656 |  |  | 10 |
| LTSC | 0.504633 | 0.078515 | 0.024829 |  |  | 10 |
| EE | 0.984513 | 0.077945 | 0.020125 |  |  | 15 |
| **Fig. 1c** |  |  |  | One way ANOVA/ Bonferroni | ***P˂0.001 |  |
| DW | 1 | 0 | 0 |  |  | 10 |
| STSC | 0.659485 | 0.059894 | 0.01894 |  |  | 10 |
| LTSC | 0.446914 | 0.059811 | 0.018914 |  |  | 10 |
| EE | 0.897099 | 0.101891 | 0.026308 |  |  | 15 |
|  |  |  |  |  |  |  |
| **Fig. 2b** |  |  |  | One way ANOVA/ Bonferroni | ***P˂0.001 |  |
| DW | 1 | 0 | 0 |  |  | 6 |
| STSC | 1.497699 | 0.183073 | 0.074739 |  |  | 6 |
| LTSC | 1.844581 | 0.263067 | 0.107396 |  |  | 6 |
| EE | 0.831435 | 0.098909 | 0.040379 |  |  | 6 |
| **Fig. 2c** |  |  |  | One way ANOVA/ Bonferroni | ***P˂0.001 |  |
| DW | 1 | 0 | 0 |  |  | 6 |
| STSC | 1.275329 | 0.164087 | 0.066988 |  |  | 6 |
| LTSC | 1.278563 | 0.195486 | 0.079807 |  |  | 6 |
| EE | 1.021737 | 0.139716 | 0.057039 |  |  | 6 |
| **Fig. 2d** |  |  |  |  |  |  |
| DW | 1 | 0 | 0 | One way ANOVA/ Bonferroni | ***P˂0.001 | 6 |
| STSC | 1.326082 | 0.095309 | 0.03890974 |  |  | 6 |
| LTSC | 1.326722 | 0.06964 | 0.028430583 |  |  | 6 |
| EE | 0.978597 | 0.070938 | 0.028960219 |  |  | 6 |
|  |  |  |  |  |  |  |
| **Fig. 2e** |  |  |  | One way ANOVA/ Bonferroni | **P˂0.01  ***P˂0.001 |  |
| DW | 1 | 0 | 0 |  |  | 6 |
| STSC | 0.948332 | 0.082078 | 0.033508 |  |  | 6 |
| LTSC | 0.758013 | 0.117007 | 0.047768 |  |  | 6 |
| EE | 1.027877 | 0.077317 | 0.031565 |  |  | 6 |
|  |  |  |  |  |  |  |
| **Fig. 3b** |  |  |  | One way ANOVA/ Bonferroni | ***P˂0.001 |  |
| DW | 1 | 0 | 0 |  |  | 6 |
| STSC | 1.236038 | 0.03812 | 1.816307 |  |  | 6 |
| LTSC | 4.670899 | 0.107433 | 0.062392 |  |  | 6 |
| EE | 1.816307 | 0.043859 | 0.025471 |  |  | 6 |
| **Fig. 3d** |  |  |  | One way ANOVA/ Bonferroni | **P˂0.01  ***P˂0.001 |  |
| DW | 1 | 0 | 0 |  |  | 6 |
| STSC | 0.603444 | 0.060327 | 0.024628 |  |  | 6 |
| LTSC | 0.386526 | 0.060159 | 0.02456 |  |  | 6 |
| EE | 1.105725 | 0.055727 | 0.02275 |  |  | 6 |
|  |  |  |  |  |  |  |
| **Fig. 4d** |  |  |  | Two Way Analysis of Variance/ Bonferroni t-test | *P˂0.05  ***P˂0.001 |  |
| DW - Dorsal | 33.66667 | 1.416569 | 0.578312 |  |  | 6 |
| STSC - Dorsal | 24.18333 | 0.92826 | 0.378961 |  |  | 6 |
| LTSC - Dorsal | 21.20833 | 1.417892 | 0.578852 |  |  | 6 |
| EE - Dorsal | 47.5 | 1.43527 | 0.585947 |  |  | 6 |
| DW - Ventral | 33.41667 | 1.428869 | 0.583333 |  |  | 6 |
| STSC - Ventral | 45.75 | 1.461164 | 0.596518 |  |  | 6 |
| LTSC - Ventral | 27.41667 | 1.428869 | 0.583333 |  |  | 6 |
| EE - Ventral | 41.75 | 3.221025 | 1.314978 |  |  | 6 |
|  |  |  |  |  |  |  |
| **Fig. 5b** |  |  |  | One way ANOVA/ Bonferroni | ***P˂0.001 |  |
| DW | 1 | 0 | 0 |  |  | 6 |
| STSC | 0.641327 | 0.037076 | 0.015136 |  |  | 6 |
| LTSC | 0.411674 | 0.020552 | 0.00839 |  |  | 6 |
| EE | 0.885276 | 0.056462 | 0.023051 |  |  | 6 |
| **Fig. 5c** |  |  |  | One way ANOVA/ Bonferroni | **P˂0.01  ***P˂0.001 |  |
| DW | 1 | 0 | 0 |  |  | 6 |
| STSC | 0.732377 | 0.020492 | 0.008365884 |  |  | 6 |
| LTSC | 0.666119 | 0.015912 | 0.006495986 |  |  | 6 |
| EE | 1.03795 | 0.045127 | 0.018422872 |  |  | 6 |
| **Fig. 5d** |  |  |  | One way ANOVA/ Bonferroni | ***P˂0.001 |  |
| DW | 1 | 0 | 0 |  |  | 6 |
| STSC | 0.385247 | 0.042681 | 0.017425 |  |  | 6 |
| LTSC | 0.31284 | 0.016562 | 0.006761 |  |  | 6 |
| EE | 1.057237 | 0.09315 | 0.038028 |  |  | 6 |
| **Fig. 5e** |  |  |  | One way ANOVA/ Bonferroni | **P˂0.01  ***P˂0.001 |  |
| DW | 1 | 0 | 0 |  |  | 6 |
| STSC | 0.244346 | 0.02847 | 0.517824 |  |  | 6 |
| LTSC | 0.282644 | 0.019736 | 0.038379 |  |  | 6 |
| EE | 0.517824 | 0.008057 | 0.015668 |  |  | 6 |
|  |  |  |  |  |  |  |
| **Fig. 6b** |  |  |  | One way ANOVA/ Bonferroni | ***P˂0.001 |  |
| DW | 1 | 0 | 0 |  |  | 6 |
| STSC | 0.595379 | 0.073049 | 0.029822137 |  |  | 6 |
| LTSC | 0.350617 | 0.105266 | 0.042974476 |  |  | 6 |
| EE | 0.741667 | 0.063226 | 0.025812064 |  |  | 6 |
| **Fig. 6c** |  |  |  |  |  |  |
| DW | 1 | 0 | 0 | One way ANOVA/ Bonferroni | *P˂0.05  ***P˂0.001 | 6 |
| STSC | 0.936032 | 0.058149 | 0.023739 |  |  | 6 |
| LTSC | 0.817903 | 0.111299 | 0.045438 |  |  | 6 |
| EE | 1.094973 | 0.093521 | 0.03818 |  |  | 6 |
|  |  |  |  |  |  |  |
| **Fig. 7b** |  |  |  |  |  |  |
| DW | 1 | 0 | 0 | One way ANOVA/ Bonferroni | *P˂0.05  ***P˂0.001 | 6 |
| STSC | 3.83859 | 0.411776 | 0.168107 |  |  | 6 |
| LTSC | 4.744362 | 0.449512 | 0.183512 |  |  | 6 |
| EE | 1.136703 | 0.135837 | 0.055455 |  |  | 6 |
| **Fig. 7c** |  |  |  | One way ANOVA/ Bonferroni | ***P˂0.001 |  |
| DW | 1 | 0 | 0 |  |  | 6 |
| STSC | 1.423465 | 0.090635 | 0.037002 |  |  | 6 |
| LTSC | 2.017716 | 0.149493 | 0.06103 |  |  | 6 |
| EE | 1.41756 | 0.090318 | 0.036872 |  |  | 6 |
| **Fig. 7d** |  |  |  | One way ANOVA/ Bonferroni | *P˂0.05 |  |
| DW | 1 | 0 | 0 |  |  | 6 |
| STSC | 1.025755 | 0.047078 | 0.019219407 |  |  | 6 |
| LTSC | 0.870773 | 0.019739 | 0.008058334 |  |  | 6 |
| EE | 0.953041 | 0.045429 | 0.018546191 |  |  | 6 |
| **Fig. 7e** |  |  |  | One way ANOVA/ Bonferroni | *P˂0.05 |  |
| DW | 1 | 0 | 0 |  |  | 6 |
| STSC | 1.003941 | 0.050996 | 0.020819 |  |  | 6 |
| LTSC | 0.780491 | 0.113564 | 0.046362 |  |  | 6 |
| EE | 0.991402 | 0.085094 | 0.034739 |  |  | 6 |
|  |  |  |  |  |  |  |
| **Fig. 8c** |  |  |  | Two Way Analysis of Variance/ Bonferroni t-test | ***P˂0.001 |  |
| DW - Feces | 1783939 | 168357 | 68731 |  |  | 6 |
| STSC - Feces | 1748663 | 127276 | 51960 |  |  | 6 |
| LTSC - Feces | 1236027 | 150829 | 61575 |  |  | 6 |
| EE - Feces | 5182020 | 216242 | 88280 |  |  | 6 |
| DW - Hippocampus | 222301 | 16732 | 6831 |  |  | 6 |
| STSC- Hippocampus | 0 | 0 | 0 |  |  | 6 |
| LTSC- Hippocampus | 0 | 0 | 0 |  |  | 6 |
| EE- Hippocampus | 71725 | 1279 | 522 |  |  | 6 |
| **Fig. 8d** |  |  |  | Two Way Analysis of Variance/ Bonferroni t-test | ***P˂0.001 |  |
| DW - Feces | 0 | 0 | 0 |  |  | 6 |
| STSC - Feces | 22494 | 1416 | 578 |  |  | 6 |
| LTSC - Feces | 29257 | 1496 | 610 |  |  | 6 |
| EE - Feces | 0 | 0 | 0 |  |  | 6 |
| DW - Hippocampus | 0 | 0 | 0 |  |  | 6 |
| STSC- Hippocampus | 5916 | 1416 | 578 |  |  | 6 |
| LTSC- Hippocampus | 6835 | 1415 | 578 |  |  | 6 |
| EE- Hippocampus | 0 | 0 | 0 |  |  | 6 |
