# Supplementary material for: Environmental enrichment improves social isolation-induced memory impairment: The possible role of ITSN1-Reelin-AMPA receptor signaling pathway
Source: PLoS One. 2024 Jan 19;19(1):e0294354. doi: 10.1371/journal.pone.0294354 (PMC10798460; doi:10.1371/journal.pone.0294354)
Supplement: Attachment — Submitted filename: Response to reviewers.docx [file pone.0294354.s002.docx]

**Journal Requirement:**

1. Please ensure that your manuscript meets PLOS ONE's style requirements, including those for file naming.

**-Yes, we have followed the PLOS ONE’s format style**

2. In your Methods section, please provide additional information regarding the permits you obtained for the collection of the field-caught rodents. Please ensure you have included the full name of the authority that approved the process and, if no permits were required, a brief statement explaining why. Please also include, in your Methods section, a statement confirming that the trapping method was specifically reviewed and approved by the IACUC

**-Yes, we have provided the details in method section of the revised manuscript Page No. 15, Line : 7-13**

3. We noticed you have some minor occurrence of overlapping text with the following previous publication(s), which needs to be addressed:

- <https://www.sciencedirect.com/science/article/abs/pii/S0016648020303944?via%3Dihub>

<https://karger.com/dne/article-abstract/44/6/547/835246/Reduced-Reelin-Expression-Induces-Memory-Deficits?redirectedFrom=fulltext>

<https://journals.plos.org/plosone/article?id=10.1371%2Fjournal.pone.0127945>

In your revision ensure you cite all your sources (including your own works), and quote or rephrase any duplicated text outside the methods section. Further consideration is dependent on these concerns being addressed

**- Above mentioned three cite are my previous publications, all the three are cited in this manuscript.**

4. Thank you for stating the following in the Acknowledgments Section of your manuscript:

"The Department of Animal Science is supported by Department of Science and Technology (DST)-Fund for Improvement of S&T Infrastructure (FIST) (DST File No. SR/FST/LSI-647/2015(G) dt.11.08.2016), Rashtriya Uchchatar Shiksha Abhiyan (RUSA) 2.0-Biological Sciences (TN.No.311/RUSA/2018 dt.12.10.2020)."

We note that you have provided additional information within the Acknowledgements Section that is not currently declared in your Funding Statement. Please note that funding information should not appear in the Acknowledgments section or other areas of your manuscript. We will only publish funding information present in the Funding Statement section of the online submission form.

Please remove any funding-related text from the manuscript and let us know how you would like to update your Funding Statement. Currently, your Funding Statement reads as follows:

"SS is recipient of Council of Scientific and Industrial Research (CSIR)-JRF Research Fellowship (File No. 09/475(0204)/2021-EMR-I). <https://newfms.ncl.res.in/>

KERKER received research grant from Department of Science & Technology (EMR/2016/005217 dt.21.03.2018)

https://www.serbonline.in/SERB/HomePage

The funders had no role in study design, data collection and analysis, decision to publish, or preparation of the manuscript."

Please include your amended statements within your cover letter; we will change the online submission form on your behalf.

**- Yes, we have corrected in the revised manuscript and the statement included in the cover letter.**

5. PLOS ONE now requires that authors provide the original uncropped and unadjusted images underlying all blot or gel results reported in a submission’s figures or Supporting Information files. This policy and the journal’s other requirements for blot/gel reporting and figure preparation are described in detail at https://journals.plos.org/plosone/s/figures#loc-blot-and-gel-reporting-requirements and <https://journals.plos.org/plosone/s/figures#loc-preparing-figures-from-image-files>. When you submit your revised manuscript, please ensure that your figures adhere fully to these guidelines and provide the original underlying images for all blot or gel data reported in your submission. See the following link for instructions on providing the original image data: <https://journals.plos.org/plosone/s/figures#loc-original-images-for-blots-and-gels>.

In your cover letter, please note whether your blot/gel image data are in Supporting Information or posted at a public data repository, provide the repository URL if relevant, and provide specific details as to which raw blot/gel images, if any, are not available. Email us at plosone@plos.org if you have any questions.

**- Yes, we have included uncropped western blot images as supplementary figure in the supplementary information.**

6. Your ethics statement should only appear in the Methods section of your manuscript. If your ethics statement is written in any section besides the Methods, please move it to the Methods section and delete it from any other section. Please ensure that your ethics statement is included in your manuscript, as the ethics statement entered into the online submission form will not be published alongside your manuscript

-**Yes, the ethical approval statement mentioned reported at the end of the method section in revised manuscript.**

7. In your Data Availability statement, you have not specified where the minimal data set underlying the results described in your manuscript can be found. PLOS defines a study's minimal data set as the underlying data used to reach the conclusions drawn in the manuscript and any additional data required to replicate the reported study findings in their entirety. All PLOS journals require that the minimal data set be made fully available. For more information about our data policy, please see <http://journals.plos.org/plosone/s/data-availability>.

Upon re-submitting your revised manuscript, please upload your study’s minimal underlying data set as either Supporting Information files or to a stable, public repository and include the relevant URLs, DOIs, or accession numbers within your revised cover letter. For a list of acceptable repositories, please see http://journals.plos.org/plosone/s/data-availability#loc-recommended-repositories. Any potentially identifying patient information must be fully anonymized.

Important: If there are ethical or legal restrictions to sharing your data publicly, please explain these restrictions in detail. Please see our guidelines for more information on what we consider unacceptable restrictions to publicly sharing data: <http://journals.plos.org/plosone/s/data-availability#loc-unacceptable-data-access-restrictions>.  Note that it is not acceptable for the authors to be the sole named individuals responsible for ensuring data access.

We will update your Data Availability statement to reflect the information you provide in your cover letter.

**- Yes, the minimal data set is provided in the supplementary information**

8. Please upload a new copy of Figures 1 and 8 as the detail is not clear. Please follow the link for more information: https://blogs.plos.org/plos/2019/06/looking-good-tips-for-creating-your-plos-figures-graphics/" <https://blogs.plos.org/plos/2019/06/looking-good-tips-for-creating-your-plos-figures-graphics/>

**- Yes, the new figures 1-8 is uploaded along the revised manuscript.**

**Response Reviewers:**

**Reviewer #1:**

1. The writing quality of this manuscript needs to improve. For instance, in the Introduction Section, the authors need introduce the published work of social isolation-induced memory impairment as well as the ameliorating effect of environmental enrichment on social isolation-induced behavioral impairment. In the Discussion Section, the authors also need compare their finding with the previous published papers.

**-Yes, in the revised manuscript we have included earlier reports in introduction section (Page No. 5, Line: 15-19) and also in the discussion section (Page No. 23, Line: 4-7 )**

2. The description of animals in groups is not clear. How many animals in each group are kept in a cage and how many cages in each group?

- **In the revised manuscript, the detailed number of animals (total number of animals captured; and final numbers taken for analysis is mentioned and number of animals housed in cage (Page No. 6, Line: 12; Page No. 7, Line: 6-16 )**

What principles are used to screen mice for behavioral, pathological, and molecular analysis?

- **In the revised manuscript we have included in the method section** **(Page No. 6, Line: 12-14; Page No. 7, Line: 18-19 )**

It is suggested that the author draw an experimental flow chart.

- **Yes, the flow chard with description included in the revised manuscript supplementary information.**
- 3. The resolution of the picture is too low. In particular, the symbols in the picture are difficult to distinguish. In addition, the layout of the pictures should be further optimized, such as Figure 3 and Figure 4 can be arranged in a page.
- **Yes, we have revised the figures as per the suggestion.**
- 4. The statistical method, the meaning of the corresponding symbol in the figure, and the number of animals in each group should be indicated in the figure legends.
- **Yes, we have increased the font size for symbol and included the number of animals in the figure legends of the revised the figures.**

5. The authors also should make clear the reasons for the selection of male animals and for observing the hippocampus.

- **Yes, we have included the statement in the discussion section of the revised manuscript (Page No. 27, Line: 16-20)**

**Reviewer #2:** This study uses wild-caught mus booduga to investigate how environmental enrichment and social isolation alter many hippocampal molecular markers linked to synaptic transmission and neuronal excitability as well as impact cognitive performance. Overall, the experiments and well-performed, the analysis and statistics are robust. Some improvements are needed for the materials and methods, as well as improvements in figure readability. Importantly, correction needs to be made for the grammar errors throughout the text.

Please provide more details about the experimental methods, specifically the caging conditions:
- What bedding was used for all of the caging conditions?

-**We have included in the details in the revised manuscript method section** **(Page No. 7, Line: 5-16)**

“How much is “plenty of food and water.” Ab libitum? Please specify

**We have used alternative to “ *ad libitum*” corrected in the revised manuscript** **(Page No. 6, Line: 15-16)**

- “Healthy” mice were used. How much did they weigh? How was health assessed?

- **In the revised manuscript we have included in the method section** **(Page No. 6, Line: 12-14; Page No. 7, Line: 18-19 )**

  Please provide more detail about the scoring and analysis of the behavioral videos. How was the timing recorded? What software was used?
- -**We have included in the details in the revised manuscript method section** **(Page No. 8, Line: 16-17)**

  Please provide a definition of measures of working memory and reference memory in the methods.
- -**We have included in the details in the revised manuscript method section** **(Page No. 8, Line: 1-3)**
- Please provide title for the figures, this will enhance readability.
- **Figure titles are included in the figure legend of the revised manuscript.**

  Figure 1 – Please provide explanation in the figure legend for the meaning of DW, STSC, LTSC and EE. Also, which test is being performed here?
- **Details are included in the revised manuscript**

  Figure 2 – the bands are very dark- in the copy I have downloaded I can barely see the SYT4 and BDNF signals over the dark background.
- **- Thank you, we have replaced the SYT4, BDNF, and other markers with new blots**
- Figure 4 – the background signal is very bright,, I cannot see the labelling well at all. Could higher-quality images please be provided? Also, the scale bars are illegible.
- **Thank you, the experiment was performed again and replaced the old images.**
- Figure 5 – same problem for figure 2.
- **Thank you, we have replaced the dark background blot with new blots**

  Figure 8 – the text in panels a and b is illegible. TOO SMALL. Please adjust the figure so that normal sized font can be used.
- **- Thank you, the larger font is used in the revised image.**
